# 3′-8″- Biflavones: A Review of Their Structural Diversity, Natural Occurrence, Role in Plants, Extraction and Identification

**DOI:** 10.3390/molecules29194634

**Published:** 2024-09-29

**Authors:** Dunja Šamec, Iva Jurčević Šangut, Erna Karalija, Bojan Šarkanj, Bruno Zelić, Anita Šalić

**Affiliations:** 1 Department of Food Technology, University North, Trg Dr. Žarka Dolinara 1, HR-48000 Koprivnica, Croatia; ijurcevic@unin.hr (I.J.Š.); bsarkanj@unin.hr (B.Š.); 2 Laboratory for Plant Physiology, Department of Biology, Faculty of Science, University of Sarajevo, Zmaja od Bosne 33-35, 71 000 Sarajevo, Bosnia and Herzegovina; erna.k@pmf.unsa.ba; 3 University of Zagreb Faculty of Chemical Engineering and Technology, Department of Reaction Engineering and Catalysis, Marulićev trg 19, HR-10000 Zagreb, Croatia; bzelic@fkit.unizg.hr; 4 Department of Packaging, Recycling and Environmental Protection, University North, Trg dr. Žarka Dolinara 1, HR-48000 Koprivnica, Croatia; 5 University of Zagreb Faculty of Chemical Engineering and Technology, Department of Thermodynamics, Mechanical Engineering and Energy, Marulićev trg 19, HR-10000 Zagreb, Croatia; asalic@fkit.unizg.hr

**Keywords:** 3′-8″-biflavones, amentoflavone, ginkgetin, extraction

## Abstract

Dimeric forms of flavonoids, known as biflavonoids, are much less studied compared to monomeric forms. It is estimated that nearly 600 different natural biflavonoids have been described to date, containing various subtypes that can be subdivided according to the position of their combinations and the nature of the subunits. The group in which two monomers are linked by a 3′-8″-C atom includes the first isolated biflavonoid ginkgetin, derivatives of amentoflavone, and several other compounds. 3′-8″-biflavones recently attracted much attention as potential molecules with biological activity such as antiviral and antimicrobial activity and as effective molecules for the treatment of neurodegenerative and metabolic diseases and in cancer therapies. With the growing interest in them as pharmacologically active molecules, there is also increasing interest in finding new natural sources of 3′-8″-biflavones and optimizing methods for their extraction and identification. Herein, we have summarized the available data on the structural diversity, natural occurrence, role in plants, extraction, and identification of 3′-8″-biflavones.

## 1. Introduction

Flavonoids are undoubtedly the best known and most studied specialized metabolites. They are produced by plants primarily through two distinct pathways: the acetate pathway (ring A) and the shikimate pathway (ring B), along with the connecting chain (ring C) that forms the C6-C3 component [1]. In a plant, they are essential for plant–environment interaction, but in science, they have come into focus as potential natural compounds to treat various diseases due to their antioxidant [2], antimicrobial [3], anti-inflammatory [4], neuroprotective [5], anticancer [6], and other activities [7]. Although some of the activities are often associated with the presence of flavonoids in general, their roles in plants and biological activity are largely dependent on the molecular structure [8]. Flavonoids include several subclasses of compounds, such as flavones, isoflavones, flavonols, flavanols, flavanones, flavanonols, chalcones and dihydrochalcones, aurones, and anthocyanidins [3], which can be further modified by glycolization, esterification, or polymerization. They can occur in free form, but in plants, plant foods, and pharmaceutical preparations, they are mostly present in conjugated form, with one or more sugar residues attached by β-glycosidic bonds to a hydroxyl group (*O*-glycosides) or a carbon atom of the aromatic ring (*C*-glycosides) [9]. Flavonoids can be polymerized, with two, three, or more monomers forming a new molecule that has a different biological activity than the monomers.

**Figure 1:**
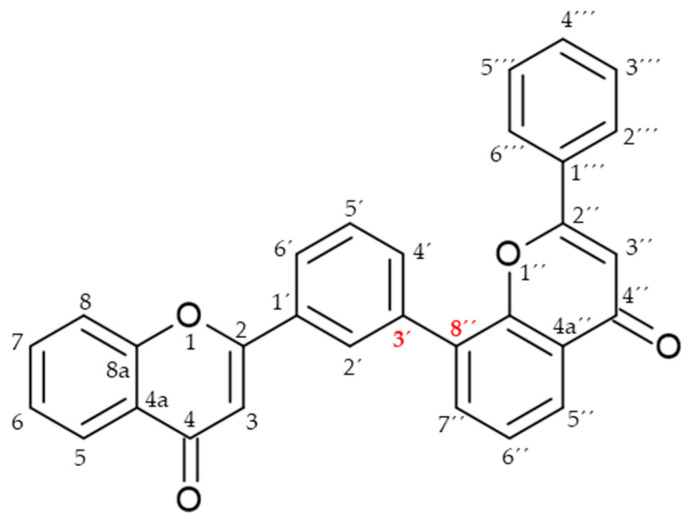
Molecular structure of 3′-8″-biflavones.

Biflavonoids or flavonoid dimers are a class of flavonoids that have been known for almost 100 years, since the first biflavonoid–ginkgetin was isolated from yellow ginkgo leaves in 1929 [10]. Biflavonoids are much less studied compared to monomeric flavonoids, although studies show a wide range of pharmacological activities, including anti-inflammatory, antioxidant, antibacterial, antiviral, antidiabetic, antitumor, cytotoxic, and neuroprotective properties [11]. According to He et al. [11] nearly 600 different biflavonoid structures have been described, which can be divided into two groups: C-C and C-linear fragments-C biflavonoids, depending on whether the linker between the two residues contains an atom. The C-C type contains different subtypes, which can be divided according to the position of their combinations into: 2-3″, 2′-2‴, 2′-6″, 2′-8″, 3-3″, 3-3‴, 3′-3‴, 3′-4‴, 3′-5″, 3-6″, 3′-6″, 3-7″, 3′-7″, 3-8″, 3′-8″, 4-6″, 4-8″, 4′-8″, 5-5″, 6-6″, 6-γ, 6-8″, 7-7″, and 8-8″. The group of biflavonoids in which two flavones are linked by a 3′-8″ C atom (Figure 1) includes the first isolated biflavonoid ginkgetin, derivatives of amentoflavone, and various other compounds that possess biological activity.

Most notably, during the coronavirus pandemic, they became known as potential antiviral agents against SARS-CoV2 viruses [12], but they also may be beneficial in the treatments of other conditions. Recently, several review papers have focused on biflavones’ antiviral and other antimicrobial activity [12,13], neurodegenerative effects [14,15,16], and roles in metabolism-related diseases and in cancer therapies [17,18]. However, to the best of our knowledge, there is no review summarizing the occurrence, possible role in plants, extraction of, or identification technique for amentoflavone and their derivatives, known as 3′-8″ -biflavones.

## 2. Structural Diversity of 3′-8″-Biflavones

Monomeric subunits of biflavones are, as the name implies, flavones a subclass of flavonoids that differ from other flavonoids in that they have a double bond between C2 and C3 in the flavonoid skeleton, there is no substitution at the C3 position, and they are oxidized at the C4 position [19] Flavones may contain various number of hydroxy group and form molecules with distinct biological activity such as chrysin (5,7-dihydroxyflavone) [20], apigenin (4′,5,7-trihydroxyflavone) [21], baicalein (5,6,7-trihydroxyflavone), luteolin (3′,4′,5,7-tetrahydroxyflavone) [22], norwogonin (5,7,8-trihydroxyflavone), tangeritin (4′,5,6,7,8-pentamethoxyflavone) [23], etc. The hydroxy group in the structure may be methylated, and *O*-methylated flavones, ones that obtain a methyl group through hydroxyl group and *C*-methylated flavonones, in which the methyl group is directly bound to C atoms of the basic skeleton, may be formed. Methylated biflavones are acacetin (5,7-dihydroxy-4′-methoxyflavone), genkwanin (4′,5-dihydroxy-7-methoxyflavone), echioidinin (5,2′-dihydroxy-7-methoxyflavone), negletein (5,6-dihydroxy-7-methoxyflavone), wogonin (5,7-dihydroxy-8-methoxyflavone), echtochrysin (5-hydroxy-7-methoxyflavone), chrysoeriol (4′,5,7-trihydroxy-3′-methoxyflavone), and many others. Methylated derivatives usually show higher bioactivity, but bioactivity depends on the position of methylated group and the number of methylated and hydroxy groups [24,25]. Another modification that affects biological activity is prenylation, which can form prenylated flavones with different biological activity, but only a few prenylated flavones have been studied in detail [26].

Flavones may be present in plant material in free form or may be glycolyzed, but also, they can form dimers at different positions. Among them, those forming dimers at 3′-8″ (Figure 1) stand out as molecules with different biological activity [15,27,28]. The formulas of the naturally occurring 3′-8″-biflavones and their methylated forms which are known to date are given in Table 1.

It should be noted that most of the known 3′-8″-biflavones were isolated and characterized 50 or more years ago, and many of them were not subsequently explored. Thus, there is a possibility that some of the compounds were inadvertently misidentified because of the lack of commercial standards and modern high-sensitivity instruments for identification at that time. In addition, the nomenclature of (bi-)flavonoids was not standardized at that time, so the same compound could be referred to in different ways, especially in the case of isomers, where isomeric structures can be referred to as the same molecule in different publications.

As can be noticed from Table 1, 3′-8″-biflavones contain two flavone subunits, and, like their monomeric subunits, may differ in a number of hydroxyl and methylated groups. For monomers, methylation is known to increase metabolic stability by preventing the formation of glucuronic acid and sulfate conjugates, resulting in increased membrane transport that facilitates absorption and greatly increases bioavailability [25]. Also, methylated monomeric derivatives usually show higher bioactivity, and the site as well as extent of methylation play an important role [24,25]. In the case of biflavonoids, including biflavones, how dimerization and the degree of methylation affect metabolism and biological activity is not yet well documented.

**Figure 2:**
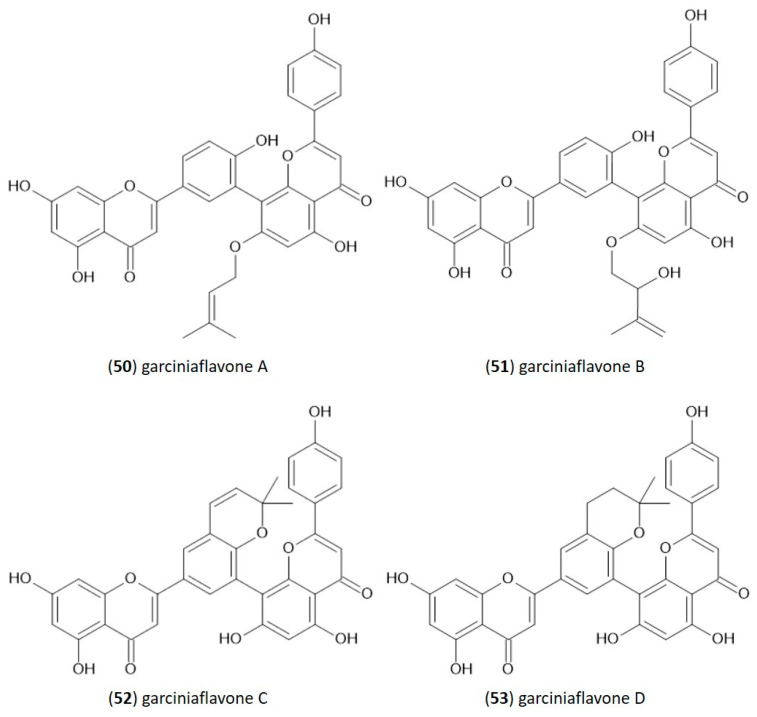
Molecular structure of naturally occurring prenylated 3′-8″-biflavones.

In the structure of 3′-8″-biflavones, the carbon–carbon double bonds C2-C3 and C2″-C3″ can be readily hydrogenated, resulting in a wide range of naturally occurring hydrogenation products (Table 2). Similarly to monomeric flavones, biflavones can also occur in a prenylated form, such as in plants of the genus *Garcinia*, from which several different prenylated 3′-8″-biflavones have been reported [29], and the structure of which is shown in Figure 2. Prenylated forms of biflavonoids are considered very rare in nature, and data have been reported only in *Garcinia* sp.

In the vast majority of reports, 3′-8″-biflavones are described as aglycones, but, for example, in whisk fern (*Psilotum nudum* L.), amentoflavone has also been detected in a glycoside form with one to three sugars attached [30,31] Amentoflavone, ginkgetin, and isoginkgetin glycosides have also been detected in ginkgo (*Ginkgo biloba* L.) (summarized by Liu et al. [32]) This shows similar behaviour to the monomeric forms, but according to the available data, the biflavone glycosides are present in much lower concentrations than the free biflavones, which is in contrast to the monomeric forms, where the glycoside forms are normally more abundant.

## 3. Distribution in the Plant Kingdom

**Figure 3:**
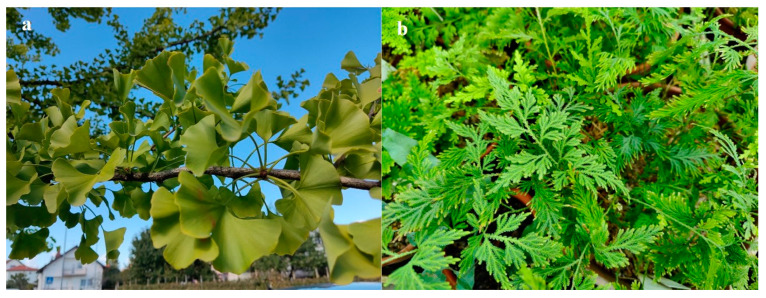
Plants abundant in 3′-8″-biflavones: (**a**) *G. biloba* L. and (**b**) *Selaginella* sp.

The first isolated 3′-8″-biflavone, and biflavonoid altogether, was isolated in 1932 as a yellow flavonoid pigment from the yellowed leaves of *G. biloba* L. (Figure 3a), and it was later named ginkgetin [33]. Isoginkgetin and bilobetin were also named after ginkgo, but several other 3′-8″-biflavones were named after the plants from which they were first isolated (Table 3).

Later, other biflavones from ginkgo leaves were also characterized [40], and to date, ginkgo is commonly mentioned and studied as a plant containing various 3′-8″-biflavones. Eight different 3′-8″ biflavone aglycones and three glycosides have been detected in ginkgo leaves [21,32], with amentoflavone, bilobetin, ginkgetin, isoginkgetin, and sciadopitysin being the most commonly detected. Older studies considered biflavonoid compounds to be characteristic of gymnosperms [41], with 3′-8″-biflavones most frequently detected in the gymnosperm families Cupressaceae, Taxaceae, and Podocarpaceae, where they occurred in 22, 16, and 12 different species (Table 4). Although most of the older studies reported their occurrence only in gymnosperms, 3′-8″-biflavons were later also found in various angiosperm families such as Euphorbiaceae, Clusiaceae, Nartheciaceae, Primulaceae, Phyllanthaceae, Oxalidaceae, Malpighiaceae, Fabaceae, Calophyllaceae, Burseraceae, Capparaceae, Salicaceae, Connaraceae, Cyperaceae, Moraceae, Putranjivaceae, Ericaceae, Hypericaceae, Lanariaceae, Caprifoliaceae, Anacardiaceae, Ranunculaceae, Thymelaeceae, Viburnaceae, and Ochnaceae. In addition, 3′-8″-biflavones were detected in pteridophytes from the families Psilotaceae and Selaginellaceae. They were particularly abundant in various spikemosses (*Selaginella* sp.) (Figure 3b, in which 71 different species of biflavonoids were detected, 24 of which were 3′-8″-biflavones represented mainly by amentoflavone and isoginkgetin [18]. The list of plants in which the presence of 3′-8″-biflavones was reported is shown in Table 4.

The accumulation of 3′-8″-biflavones is highly dependent on the tissue type studied and other environmental conditions. For example, ginkgo leaves are rich in bilobetin, isoginkgetin, ginkgetin, and sciadopytisin [177,178], but their content is highly dependent on the growing location of the ginkgo plant [179] and the developmental stage of the leaves [177,178], so these parameters should be taken into account when ginkgo leaves are used as a source of 3′-8″-biflavones for pharmaceutical purposes. According to currently available data, the yellow autumn leaves are more abundant in 3′-8″-biflavones than the green leaves used in traditional medicine and for extract preparations [27]. In addition to the leaves, 3′-8″-biflavones have also been detected in other parts of the ginkgo plant, but their content depends strongly on the tissue type, with the leaves having the highest content, followed by the sarcotesta [178].

## 4. Role in Plants

In general, flavonoids in plants have a protective function against biotic and abiotic stress conditions. They accumulate when plants are exposed to UV-B radiation and act as sunscreens due to their absorption in the UV range. They also act as scavengers of reactive oxygen species (ROS) due to the phenolic hydroxyl groups in their structure and are often accumulated in plants exposed to various stress factors [177]. However, flavonoids are a large group of molecules that have 6000 different structures and, depending on their structure, can play different roles in plants growth, development, and protection from stress [27]. Although flavonoids are often considered as good antioxidants, biflavonoids, including 3′-8″-biflavones, have significantly lower antioxidant activity than monomeric flavonoids [136], and their role in plants is probably different from that of monomeric flavonoids.

The role of biflavonoids in plants is still largely unexplored. Their localization in plant tissues may indicate roles in plant–environment interactions. Tissue-specific profiling of five 3′-8″-biflavones, amentoflavone, bilobetin, ginkgetin, isoginkgetin, and sciadopitysin, in ginkgo showed that they are accumulated only in plant parts that are in direct contact with the environment [178]. A similar result was observed in MALDI imaging studies. Li et al. [180] used MALDI imaging to investigate the spatio-chemical localization of metabolites in ginkgo leaves and found that the 3′-8″-biflavones amentoflavone, bilobetin/sequioflavone, isoginkgetin/ginkgetin, sciadopytisyn, and methoxybilobetin accumulate in the upper and lower epidermis. The accumulation of ginkgetin/isoginkgetin on the surfaces of the ginkgo leaves was also shown by Beck and Stengel [179]. They reported an increased concentration on the lower side of the leaf compared to the upper side, which might be related to the proposed functions of biflavonoids in plants as fungitoxins and predators, because for both fungi and insects, the lower side of the leaf seems to be a preferred site of invasion. Amentoflavone in whisk fern is also accumulated in the outer part of the above-ground rhizome, according to MALDI imaging [31].

This accumulation of 3′-8″-biflavones, as we already mentioned, might be related to defence against biotic stress. 3′-8″-biflavones showed strong antimicrobial effects against pathogenic fungi in several studies. Krauze-Baranowska and Witwart [103] studied the antifungal activity of bilobetin, 4‴-*O*-methylamentoflavone, amentoflavone, 7-*O*-methylamentoflavone, ginkgetin, sciadopitysin, and 2,3-dihydrosciadopitysin against the fungi *Alternaria alternata*, *Fusarium culmorum,* and *Cladosporium oxysporum.* Bilobetin completely inhibited the growth of *C. oxysporum* and *F. culmorum* at a concentration of 100 mmol/L, but the activity of ginkgetin and 7-*O*-methylamentoflavone towards *A. alternata* was stronger than that of bilobetin. Amentoflavone, along with other biflavonoids, has been shown to affect the production of aflatoxins in *Aspergillus flavus* and *A. parasiticus* [181]. In this study, the authors found that biflavonoids were generally more active in inhibiting aflatoxin production at lower concentrations than the monomeric flavonoids, which may indicate that the dimeric structure would cause stronger activity. According to the authors [181], biflavonoids can be used to develop compounds to control aflatoxin production.

There is evidence that the role of biflavonoids in plants may be related to their role in photosynthesis, more precisely in inhibiting photosynthesis. Aguilar et al. [182] reported in their study with spinach chloroplasts that biflavonoids isolated from *Selaginella lepidophylla* inhibited ATP synthesis and several other photosynthetic processes, including electron flow, PSII, PSI, and their partial reactions on chloroplasts. Céspedes et al. [183] also reported that biflavonoids can case a concentration-dependent inhibition of photophosphorylation. In an experiment with cyanobacteria, *Microcystis aeruginosa*, a harmful cyanobacterial bloom, lost its original shape and chlorophylls after treatment with extracts containing high levels of amentoflavone [184]. In this study, the authors show that amentoflavone selectively kills only *M. aeruginosa* strains without harming other non-cyanobacteria, which may be related to the photosynthetic capacity of cyanobacteria. Few studies have also shown that amentoflavone has an allelopathic effect. De Almeida et al. [185] studied the in vitro effects of *Byrsonima crassa* extract, rich in amentoflavone, on tomato seedlings and showed that all doses tested had stimulatory effects on root length and inhibitory effects on the length of the aboveground parts of the tomato. Interestingly, biflavonoids are used as taxonomic markers in species of *Ochnaceae*, known to exhibit allelopathic activity against *Lactuca sativa* [186]. However, the exact mechanisms of action are unknown, and further studies should explain the above effects and clarify the role of 3′-8″-biflavones and biflavonoids in plants as a whole.

## 5. Extraction

As mentioned earlier, biflavonoids are much less studied compared to monomeric flavonoids, and most of the older work dealing with biflavonoids merely reports the presence of individual 3′-8″-biflavones in plant material without optimizing extraction or identification/quantification methods. As biflavones, 3′-8″-biflavones are of interest for industrial application, and progress has also been made in the development of extraction methods. Because of their beneficial properties, especially when it comes to medical and food applications, effective, controlled, and safe extraction methods are needed [187]. Before extraction, plant samples typically undergo freeze drying, convection drying, or microwave vacuum drying, followed by milling, grinding, and homogenization, after which an appropriate solvent and extraction method are selected [188,189]. Traditionally, most extraction procedures for 3′-8″-biflavones extraction are based on conventional methods such as organic solvent extraction, reflux extraction, percolation extraction, and Soxhlet extraction [104] (Table 5). Although these methods are widely used, they are time- and energy-consuming, inefficient [190,191,192] and require large volumes of possibly toxic solvents [192,193]. New solvents, ionic liquids (IL), and deep eutectic solvents (DES) are being researched more and more [191,192,193]. The main advantages of DESs are their versatility, tunability, wide temperature range, high polarity, low vapor pressure, non-flammability, and potential as eco-friendly solvents that reduce extraction costs, environmental impact, and degradation of temperature-sensitive molecules [193].

**Figure 4:**
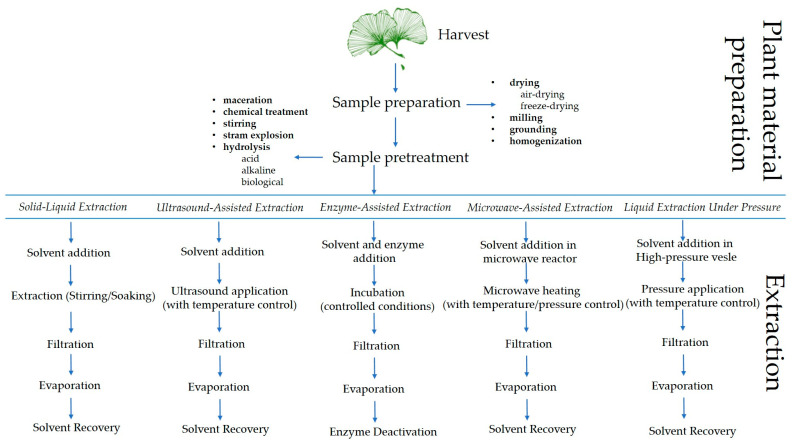
Schematic representation of the biflavonoid extraction process from plant material, highlighting key extraction steps.

To develop a successful extraction method, formulation and optimization, evaluation, and standardization of process variables are required. This is the only way to achieve a reproducible and efficient extraction process [192]. When it comes to extraction processes, the choice of solvent, liquid/solid ratio, temperature, extraction time, and plant material size are likely to be the starting point for process design and optimization [188]. When using novel methods such as ultrasound-assisted extraction (UAE), enzyme-assisted extraction (EAE), microwave-assisted extraction (MAE), and liquid extraction under pressure (PLE), some additional variables such as ultrasound frequency and power, microwave power, solvent amount, pressure, etc., should be considered [188]. To find the optimal conditions, it is necessary to optimize each process individually due to the different characteristics of biflavonoids and the sources [192,193,194,195]. Figure 4 illustrates the biflavonoid extraction process from plant material, highlighting the various steps involved in the procedure.

**Figure 5:**
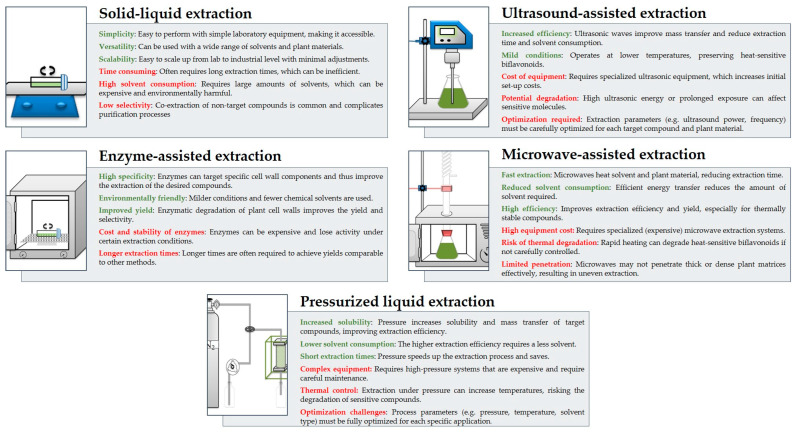
Comparison of extraction methods used for biflavonoids—advantages (green) and disadvantages (red).

The most effective extraction methods often involve a combination of several approaches, as each method has its own advantages and disadvantages, as illustrated in Figure 5.

The UAE-DES extraction process has the advantages such as short extraction time, low solvent and energy consumption, and high extraction efficiency [193]. In the paper presented by Liu et al. [196], the authors described the UAE-DES extraction of total biflavonoids (including heveaflavone and amentoflavone) from *S. chaetoloma.* Comparing the effect of UAE-DES extraction with conventional methods (maceration and percloration with 95% ethanol), the authors observed that by using UAE-DES, the extraction rate increased by 1.5–3-fold compared to conventional methods. In the paper by Li et al. [193] the authors combined MAE-IL for the extraction of amentoflavone (and hinokiflavone) from *S. sinensis*. Under optimal conditions, the content of amentoflavone was 1.96 mg/g dry weight. Compared with the conventional extraction methods, MAE-IL achieved a higher yield in a shorter time, but also reduced the consumption of the solvent. Lei et al. [188] extracted four main biflavonoids (bilobetin, ginkgetin, isoginkgetin, and sciadopitysin) from *G. biloba* L. using UAE-IL. Compared with UAE-ethanol, infiltration extraction, and percolation extraction, by applying UAE-IL, more biflavonoids were obtained in less time. In addition, the results of the recovery test indicated that the recovered IL could be repeatedly extracted six times. A comparison of different methods for 3′-8″-biflavonoid extraction is given in the Table 5.

Once the extraction is performed, unfortunately, 3′-8″-biflavonoids are not the only components present in the extract. Since neither of mentioned methods is selective to extract only 3′-8″-biflavonids, the extract is a mixture of different phytochemicals. Due to this, and in order for 3′-8″-biflavonoids to be used in pharmacology, they need to be isolated with high purity. The most common methods used for 3′-8″-biflavonoid isolation are liquid–liquid extraction, macroporous resin adsorption, antisolvent crystallization, and chromatography, with chromatography being the primary technique to obtain high-purity compounds. Column chromatography, using silica gel, polyamide, and sephadex LH 20 as packing materials, has been successfully employed for this purpose. Although this method is widespread, it is time-consuming, expensive, and not environmentally friendly since it requires large amounts of organic solvents. To obtain high purity, this method has to be repeated multiple times, thus leading to low recovery. The additional problem is the selectivity of traditional chromatography methods. Isolating isomers such as ginkgetin and isoginkgetin from *G. biloba* L. poses even greater challenges with traditional column chromatography [197]. As a possible solution, two-dimensional preparative HPLC methods have been proposed and applied for isolating high-purity compounds on a large scale [198]. But, as for the extraction methods, the combination of approaches is also crucial for efficient isolation of biflavonoids. In the paper presented by Shen et al. [199] the authors proposed an efficient and industrially viable protocol for large-scale targeted isolation of high-purity bioactive biflavonoids from industrial waste *G. biloba* L. exocarp. The process involved several key steps to achieve high purity and substantial yields. Firstly, macroporous adsorption resin was employed to enrich the bioflavonoids from the *G. biloba* L. waste. This step ensured the concentration of the target compounds for further processing. Next, a targeted *on-line* recognition method based on their characteristic UV absorption at 210 nm, 270 nm, and 330 nm was applied to identify and isolate the biflavonoids selectively. This recognition process facilitated the efficient separation of the desired compounds from the mixture. The core technique used in the protocol was the two-dimensional preparative normal phase/reversed phase HPLC-DAC system. This state-of-the-art system allowed for the precise and reliable isolation of the biflavonoids. Within a remarkably short period of 30 min, a total of three biflavonoids, namely, bilobetin, ginkgetin, and isoginkgetin, were isolated with purity exceeding 99.0%. The yield from each isolation run reached dozens or even hundreds of milligrams, making it highly suitable for large-scale production.

**Table 5:** Comparison of different extraction methods for 3′-8″ biflavone extraction.

	Source		Conventional Method			Novel Methods			Reference
Biflavonoid			Extraction Conditions		Yield mg/g	Extraction Conditions		Yield mg/g	
Amentoflavone	*T. chinensis* leaves		Soxhlet extractor, methanol, *t*_extraction_ = 7 h		4.08 ± 0.03	Supercritical CO_2_ extraction plus co-solvent (78% ethanol), *t*_extraction_ = 2 h, *T* = 48 °C, *p* = 25 Mpa, *q*_CO2_ = 2 L/min		4.47 ± 0.06	[104]
	*G. biloba* L.	tree bark	Sonification, *t*_sonification_ = 10 min; 80% methanol, *t*_extraction_ = 45 min, *T* = 25 °C		0.06 ± 0.004	-		-	[178]
		twig bark			0.08 ± 0.007	-		-	
		buds			0.04 ± 0.002	-		-	
		leaf blades			0.09 ± 0.001	-		-	
		petioles			0.18 ± 0.005	-		-	
		seed petioles			0.03 ± 0.002	-		-	
		sarcotesta			0.02 ± 0.002	-		-	
	*S. tamariscina* (Beauv) Spring		Solvent extraction, sonification, *t*_extraction_ = 2 h, *T* = 25 °C	70% ethanol	14.05	Supercritical CO_2_ fluid extraction extractor *T* = 60 °C, *p* = 200 bar, static, *t*_extraction static_ = 0.5 h, *t*_extraction dynamic_ = 1 h, 70% ethanol		20.18	[200]
				70% hexane	0.40				
				70% n-butanol	1.72	Accelerated solvent extraction, 70% ethanol, *t*_extraction_ = 4 min, elution is flushed with 60% volume, the nitrogen purge lasts 60 s, and extraction is performed three times. The extraction *T* = 80 °C, and *p* < 1500 psi		27.77	
				70% ethyl acetate	1.71				
			Reflux extraction, 70% ethanol, *t*_extraction_ = 1 h, *T* = 90 °C		33.00				
			Percolation extraction, *t*_extraction_ = 2 h, *T* = 40 °C		14.73				
	*S. uncinata*		Maceration extraction, DES, *t*_extraction_ = 3 h		0.05 ± 0.01	Ultrasonic-assisted deep eutectic solvent extraction, 33% (*w*/*w*), *t*_extraction_ = 0.5 h		0.71 ± 0.01	[201]
			Percolation extraction, *t*_extraction_ = 4 h, *T* = 40 °C		0.60 ± 0.01				
	*G. biloba* L. leaves		Sonification, *t*_sonification_ = 10 min; 70% ethanol, *t*_extraction_ = 45 min, *T* = 25 °C		0.064 ± 0.004	Enzyme-assisted extraction(Viscozyme L), *t*_extraction_ = 4 h, *T* = 50 °C and 200 rpm		0.066 ± 0.003	[202]
						Enzyme-assisted extraction (Viscozyme L), *t*_extraction_ = 24 h, *T* = 50 °C and 200 rpm		0.069 ± 0.002	
						Ultrasound-assisted extraction, 20 kHz, 62% amplitude, *t*_extraction_ = 10 min, *T* = 0 °C		0.064 ± 0.000	
						Mechanically assisted extraction, *t*_extraction_ = 20 min, *T* = 25 °C and 600 rpm		0.065 ± 0.001	
						Chemically assisted extraction, 0.1% TritonX and 10% NaClO solution, *T* = 25 °C and 200 rpm.		0.044 ± 0.001	
	*G. biloba* L. leaves		Sonification, *t*_sonification_ = 10 min; 80% methanol, *t*_extraction_ = 45 min, *T* = 25 °C		0.081 ± 0.002	Sonification, *t*_sonification_ = 10 min; DES, *t*_extraction_ = 45 min, *T* = 25 °C	Betaine: ethylene glycol 1:2 with 20% H_2_O (*w*/*w*)	0.061 ± 0.009	[178]
							Betaine: ethylene glycol 1:2 with 30% H_2_O (*w*/*w*)	0.053 ± 0.000	
Bilobetin	*G. biloba* L. leaves		Ethanol-based Ultrasound Assisted Extraction, 70% ethanol *t*_extraction_ = 25 min, solid–liquid ratio of 1:14 g/mL, and ultrasonic power of 280 W		2.00 *	Ultrasonic-assisted ionic liquid extraction *c*_[Epy]BF4_ = 0.148 mol/L,*t*_extraction_ = 25 min, solid–liquid ratio of 1:14 g/mL, and ultrasonic power of 280 W		2.44	[188]
			Infiltration extraction *c*_[Epy]BF4_ = 0.148 mol/L, *t*_extraction_ = 48 h		1.60 *				
			Percolation extraction *c*_[Epy]BF4_ = 0.148 mol/L, *t*_extraction_ = 30 min, percolate: *q* = 2 drops/min		1.40 *				
	*G. biloba* L.	twig bark	Sonification, *t* _sonification_ = 10 min); 80% methanol, *t*_extraction_ = 45 min, *T* = 25 °C		0.03 ± 0.002	-		-	[178]
		petioles			0.98 ± 0.006	-		-	
		leaf blades			1.38 ± 0.01	-		-	
		seed petioles			0.25 ± 0.02	-		-	
		sarcotesta			0.14 ± 0.05	-		-	
	*G. biloba* L. leaves		Sonification, *t* _sonification_ = 10 min; 70% ethanol, *t*_extraction_ = 45 min, *T* = 25 °C		0.164 ± 0.014	Enzyme-assisted extraction (Viscozyme L), *t*_extraction_ = 4 h, *T* = 50 °C, and 200 rpm		0.166 ± 0.003	[202]
						Enzyme-assisted extraction (Viscozyme L), *t*_extraction_ = 24 h, *T* = 50 °C, and 200 rpm		0.172 ± 0.002	
						Ultrasound-assisted extraction, 20 kHz, 62% amplitude, *t*_extraction_ = 10 min, *T* = 0 °C		0.167 ± 0.001	
						Mechanically assisted extraction, *t*_extraction_ = 20 min, *T* = 25 °C, and 600 rpm		0.177 ± 0.012	
						Chemically assisted extraction, 0.1% TritonX and 10% NaClO solution, *T* = 25 °C, and 200 rpm.		0.108 ± 0.023	
	*G. biloba* L. leaves		Sonification, *t*_sonification_ = 10 min; 80% methanol, *t*_extraction_ = 45 min, *T* = 25 °C		0.471 ± 0.013	Sonification, *t*_sonification_ = 10 min; DES, *t*_extraction_ = 45 min, *T* = 25 °C	Betaine: ethylene glycol 1:2 with 10% H_2_O (*w*/*w*)	0.107 ± 0.008	[203]
							Betaine: ethylene glycol 1:2 with 20% H_2_O (*w*/*w*)	0.171 ± 0.029	
							Betaine: ethylene glycol 1:2 with 30% H_2_O (*w*/*w*)	0.118 ± 0.013	
							Betaine: sucrose 1:4 with 30% H_2_O (*w*/*w*)	0.063 ± 0.000	
							Betaine: glycerol 1:2 with 10% H_2_O (*w*/*w*)	0.092 ± 0.013	
							Choline chloride: ethylene glycol 1:2 with 10% H_2_O (*w*/*w*)	0.065 ± 0.002	
							Choline chloride: ethylene glycol 1:2 with 20% H_2_O (*w*/*w*)	0.072 ± 0.000	
							Choline chloride: urea 1:2 with 10% H_2_O (*w*/*w*)	0.066 ± 0.003	
							Choline chloride: urea: ethylene glycol 1:2:2 with 10% H_2_O (*w*/*w*)	0.077 ± 0.003	
Ginkgetin	*T. chinensis* leaves		Soxhlet extractor; extraction solvent, methanol; *t*_extraction_ = 7 h		2.17 ± 0.02	Supercritical CO_2_ extraction Plus co-solvent (78% ethanol) *t*_extraction_ = 2 h, *T* = 48 °C, *p* = 25 Mpa; *q*_CO2_ = 2 L/min		3.39 ± 0.02	[104]
	*G. biloba* L. leaves		Ethanol-based ultrasound-assisted extraction, 70% ethanol *t*_extraction_ = 25 min, solid–liquid ratio of 1:14 g/mL, and ultrasonic power of 280 W		3.90 *	Ultrasonic-assisted ionic liquid extraction *c*_[Epy]BF4_ = 0.148 mol/L, *t*_extraction_ = 25 min, solid–liquid ratio of 1:14 g/mL, and ultrasonic power of 280 W		4.33	[183]
			Infiltration extraction *c*_[Epy]BF4_ = 0.148 mol/L, *t*_extraction_ = 48 h		2.60 *				
			Percolation extraction *c*_[Epy]BF4_ = 0.148 mol/L, *t*_extraction_ = 30 min, percolate: *q* = 2 drops/min		2.00 *				
	*G. biloba* L.	twig bark	Sonification, *t* _sonification_ = 10 min, 80% methanol, *t*_extraction_ = 45 min, *T* = 25 °C		0.03 ± 0.002	-		-	[178]
		buds			0.01 ± 0.001	-		-	
		petioles			0.63 ± 0.003				
		leaf blades			1.33 ± 0.005	-		-	
		seed petioles			0.15 ± 0.01	-		-	
		sarcotesta			0.12 ± 0.007	-		-	
	*G. biloba* L. leaves		Sonification, *t* _sonification_ = 10 min; 70% ethanol, *t*_extraction_ = 45 min, *T* = 25 °C		0.607 ± 0.050	Enzyme-assisted extraction (Viscozyme L), *t*_extraction_ = 4 h, *T* = 50 °C, and 200 rpm		0.627 ± 0.010	[202]
						Enzyme-assisted extraction (Viscozyme L), *t*_extraction_ = 24 h, *T* = 50 °C, and 200 rpm		0.646± 0.007	
						Ultrasound-assisted extraction, 20 kHz, 62% amplitude, *t*_extraction_ = 10 min, *T* = 0 °C		0.622 ± 0.003	
						Mechanically assisted extraction, *t*_extraction_ = 20 min, *T* = 25 °C, and 600 rpm		0.634 ± 0.009	
						Chemically assisted extraction, 0.1% TritonX and 10% NaClO solution, *T* = 25 °C, and 200 rpm.		0.466 ± 0.055	
	*G. biloba* L. leaves		Sonification, *t*_sonification_ = 10 min; 80% methanol, *t*_extraction_ = 45 min, *T* = 25 °C		0.367 ± 0.004	Sonification, *t*_sonification_ = 10 min; DES, *t*_extraction_ = 45 min, *T* = 25 °C	Betaine: ethylene glycol 1:2 with 10% H_2_O (*w*/*w*)	0.110 ± 0.010	[203]
							Betaine: ethylene glycol 1:2 with 20% H_2_O (*w*/*w*)	0.105 ± 0.016	
							Betaine: ethylene glycol 1:2 with 30% H_2_O (*w*/*w*)	0.074 ± 0.03	
							Betaine: glycerol 1:2 with 10% H_2_O (*w*/*w*)	0.073 ± 0.004	
Isoginkgetin	*G. biloba* L. leaves		Ethanol-based ultrasound-assisted extraction, 70% ethanol *t*_extraction_ = 25 min, solid–liquid ratio of 1:14 g/mL, and ultrasonic power of 280 W		5.20 *	Ultrasonic-assisted ionic liquid extraction *c*_[Epy]BF4_ = 0.148 mol/L, *t*_extraction_ = 25 min, solid–liquid ratio of 1:14 g/mL, and ultrasonic power of 280 W		6.50	[188]
			Infiltration extraction *c*_[Epy]BF4_ = 0.148 mol/L, *t*_extraction_ = 48 h		5.00 *				
			Percolation extraction *c*_[Epy]BF4_ = 0.148 mol/L, *t*_extraction_ = 30 min, percolate: *q* = 2 drops/min		3.50 *				
	*G. biloba* L.	twig bark	Sonification, *t* _sonification_ = 10 min, 80% methanol, *t*_extraction_ = 45 min, *T* = 25 °C		0.03 ± 0.003	-		-	[178]
		buds			0.005 ± 0.001	-		-	
		petioles			0.88 ± 0.005	-		-	
		leaf blades			1.90 ± 0.01	-		-	
		seed petioles			0.38 ± 0.03	-		-	
		sarcotesta			0.31 ± 0.02	-		-	
	*G. biloba* L. leaves		Sonification, *t* _sonification_ = 10 min; 70% ethanol, *t*_extraction_ = 45 min, *T* = 25 °C		0.945 ± 0.090	Enzyme-assisted extraction (Viscozyme L), *t*_extraction_ = 4 h, *T* = 50 °C, and 200 rpm		0.974 ± 0.018	[202]
						Enzyme-assisted extraction (Viscozyme L), *t*_extraction_ = 24 h, *T* = 50 °C, and 200 rpm		1.007 ± 0.013	
						Ultrasound-assisted extraction, 20 kHz, 62% amplitude, *t*_extraction_ = 10 min, *T* = 0 °C		0.969 ± 0.004	
						Mechanically assisted extraction, *t*_extraction_ = 20 min, *T* = 25 °C and 600 rpm		0.994 ± 0.015	
						Chemically assisted extraction, 0.1% TritonX and 10% NaClO solution, *T* = 25 °C, and 200 rpm.		0.631 ± 0.123	
	*G. biloba* L. leaves		Sonification, *t*_sonification_ = 10 min; 80% methanol, *t*_extraction_ = 45 min, *T* = 25 °C		0.543 ± 0.005	Sonification, *t*_sonification_ = 10 min; DES, *t*_extraction_ = 45 min, *T* = 25 °C	Betaine: ethylene glycol 1:2 with 10% H_2_O (*w*/*w*)	0.146 ± 0.016	[203]
							Betaine: ethylene glycol 1:2 with 20% H_2_O (*w*/*w*)	0.124± 0.006	
							Betaine: ethylene glycol 1:2 with 30% H_2_O (*w*/*w*)	0.094 ± 0.006	
							Betaine: sucrose 1:4 with 30% H_2_O (*w*/*w*)	0.063 ± 0.001	
							Betaine: glycerol 1:2 with 10% H_2_O (*w*/*w*)	0.082 ± 0.009	
							Choline chloride: ethylene glycol 1:2 with 10% H_2_O (*w*/*w*)	0.062 ± 0.001	
							Choline chloride: ethylene glycol 1:2 with 20% H_2_O (*w*/*w*)	0.061 ± 0.000	
							Choline chloride: urea 1:2 with 10% H_2_O (*w*/*w*)	0.061 ± 0.000	
							Choline chloride: urea: ethylene glycol 1:2:2 with 10% H_2_O (*w*/*w*)	0.062 ± 0.001	
	*G. biloba* L. leaves		Ethanol-based ultrasound-assisted extraction, 70% ethanol *t*_extraction_ = 25 min, solid–liquid ratio of 1:14 g/mL, and ultrasonic power of 280 W		10.00 *	Ultrasonic-assisted ionic liquid extraction *c*_[Epy]BF4_ = 0.148 mol/L, *t*_extraction_ = 25 min, solid–liquid ratio of 1:14 g/mL, and ultrasonic power of 280 W		13.97	[188]
			Infiltration extraction *c*_[Epy]BF4_ = 0.148 mol/L, *t*_extraction_ = 48 h		9.10 *				
			Percolation extraction *c*_[Epy]BF4_ = 0.148 mol/L, *t*_extraction_ = 30 min, percolate: *q* = 2 drops/min		9.00 *				
	*G. biloba* L.	twig bark	Sonification, *t*_sonification_ = 10 min, 80% methanol, *t*_extraction_ = 45 min, *T* = 25 °C		0.04 ± 0.004	-		-	[178]
		buns			0.01 ± 0.003	-		-	
		petioles			0.73 ± 0.003	-		-	
		leaf blades			2.40 ± 0.006	-		-	
		seed petioles			0.29 ± 0.02				
		sarcotesta			0.22 ± 0.004	-		-	
	*G. biloba* L. leaves		Sonification, *t*_sonification_ = 10 min; 70% ethanol, *t*_extraction_ = 45 min, *T* = 25 °C		1.387 ± 0.105	Enzyme-assisted extraction (Viscozyme L), *t*_extraction_ = 4 h, *T* = 50 °C, and 200 rpm		1.430 ± 0.021	[203]
						Enzyme-assisted extraction (Viscozyme L), *t*_extraction_ = 24 h, *T* = 50 °C, and 200 rpm		1.461 ± 0.105	
						Ultrasound-assisted extraction, 20 kHz, 62% amplitude, *t*_extraction_ = 10 min, *T* = 0 °C		1.419 ± 0.006	
						Mechanically assisted extraction, *t*_extraction_ = 20 min, *T* = 25 °C, and 600 rpm		1.450 ± 0.018	
						Chemically assisted extraction, 0.1% TritonX and 10% NaClO solution, *T* = 25 °C, and 200 rpm.		1.054 ± 0.099	
	*G. biloba* L. leaves		Sonification, *t*_sonification_ = 10 min; 80% methanol, *t*_extraction_ = 45 min, *T* = 25 °C		0.344 ± 0.026	Sonification, *t*_sonification_ = 10 min; DES, *t*_extraction_ = 45 min, *T* = 25 °C	Betaine: ethylene glycol 1:2 with 10% H_2_O (*w*/*w*)	0.154 ± 0.019	[203]
							Betaine: ethylene glycol 1:2 with 20% H_2_O (*w*/*w*)	0.077± 0.001	
							Betaine: ethylene glycol 1:2 with 30% H_2_O (*w*/*w*)	0.071 ± 0.005	
							Betaine: sucrose 1:4 with 30% H_2_O (*w*/*w*)	0.054 ± 0.002	
							Betaine: glycerol 1:2 with 10% H_2_O (*w*/*w*)	0.059 ± 0.001	
							Choline chloride: ethylene glycol 1:2 with 10% H_2_O (*w*/*w*)	0.051 ± 0.002	
							Choline chloride: ethylene glycol 1:2 with 20% H_2_O (*w*/*w*)	0.050 ± 0.000	
							Choline chloride: urea 1:2 with 10% H_2_O (*w*/*w*)	0.050 ± 0.000	
							Choline chloride: urea: ethylene glycol 1:2:2 with 10% H_2_O (*w*/*w*)	0.050 ± 0.001	
**Total biflavonoids**									
Amentoflavone, ginkgetin, hinokiflavone and heveaflavone	*S. helvetica*		Ethanol-based ultrasound-assisted extraction, 95% ethanol, ultrasonic power 250 W,*T* = 45 °C, *t*_extraction_ = 40 min		11.00 *	Ultrasonic-assisted ionic liquid extraction *c*_[C6mim]PF6_ = 0.78 mol/L, *t*_extraction_ = 40 min, solid–liquid ratio of 1:12.72 g/mL, and ultrasonic power of 250 W, *T* = 47.27 °C		18.69	[190]
			Heat-reflux extraction, 95% ethanol, *t*_extraction_ = 120 min		6.50 *				
			Soxhelt extraction, 95% ethanol, *t*_extraction_ = 120 min		7.00 *				
			Percolation extraction, 95% ethanol, *t*_extraction_ = 24 min		10.00 *				
Amentoflavone, robustaflavone, and hinokiflavone	*S. doederleinii*		Soxhlet extraction, 70% ethanol, *t*_extraction_ = 2 h, *T* = 95 °C		4.97 ± 0.08	Microwave-assisted extraction; 70% ethanol, 460 W microwave power, *T* = 45 °C, *t*_extraction_ = 45 min		8.91 ± 0.13	[193]
						Ionic liquid microwave-assisted extraction, *c*_(Hmim) (PF6)_ = 2 mmol/L, solvent–material ratio = 1:15 g/mL, microwave power 460 W, *T* = 45 °C, *t*_extraction_ = 40 min		16.83 ± 1.51	
Myricitrin, isoquercitrin, quercitrin, amentoflavone and hinokiflavone	*P. cacumen*		-		-	Deep eutectic solvents (choline chloride:1,4-butanediol-lactic acid 1:3) and ultrasonic extraction, ultrasonic time: 60 min, liquid/solid ratio: 20:1, and water content: 35%		23.11 ± 0.35	[204]

* Most Abundant.

## 6. Identification, Quantification, and Localization within Tissue

Progress in developing methods to identify and quantify some natural compounds depends on several factors, such as whether a compound is recognized as having biological activity, whether regulatory agencies require control of the amount in a product, or whether appropriate tools are simply available. If standards are not commercially available, identification is usually performed by NMR. In most studies reporting 3′-8″-biflavones for the first time, such as those in a Table 1, identification is performed using NMR. In the ^1^H NMR spectrum, aromatic proton signals typically appear between 6.0–8.0 ppm, while hydroxyl protons resonate as broad singlets around 10.0–12.0 ppm. ^13^C NMR spectra display aromatic carbon signals between 100 and 160 ppm, with carbonyl carbons around 175–180 ppm [43]. Carbons involved in the 3′-8″ linkage, such as C3′ and C8″, generally exhibit downfield shifts compared to non-linked carbons [43]. Due to the dimeric nature of 3′-8″-biflavones, duplicate or split signals may occur, reflecting the slightly different chemical environments of the two flavonoid units, especially near the linkage [205]. NMR spectroscopy of dimeric flavonoids is often complicated by hindered rotation of the monomers around the C–C axis (atropisomerism), leading to high spectral complexity. Several approaches have been proposed to accelerate identification, such as 1,1-ADEQUATE [206], while two-dimensional NMR techniques (HSQC, HMBC, and COSY) may help in resolving duplicate signals and confirming structural connectivity [206]. However, these techniques have not found widespread application in studies of 3′-8″-biflavones. Problems with the low sensitivity of NMR compared to other spectroscopic techniques are well known and pose a significant challenge in identifying biflavonoids, which are often present at low concentrations [205]. Over the past few decades, most advances in NMR spectroscopy have focused on increasing sensitivity. However, an even greater challenge remains: the lack of a comprehensive NMR database for the identification of 3′-8′- biflavones [205]. Many NMR databases lack information on these compounds, but the Spektraris database [207] stands out as an exception, having integrated data on biflavones and having been successfully used for the identification of biflavonoids in *P. nudum* [31]. However, the structure of some possible methyl-biflavones reported earlier should be corrected because structural elucidation in the 1960s and 1970s was based on co-chromatography with isolated authentic compounds, which may lead to misidentification [208].

**Figure 6:**
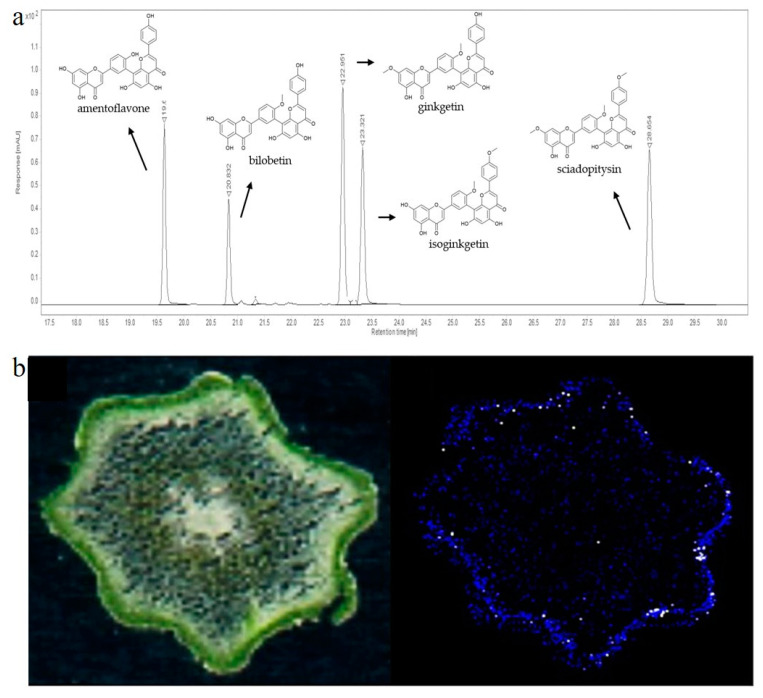
(**a**) Representative HPLC-DAD chromatogram of five biflavones recorded at 330 nm [179]; (**b**) MALDI-MS imaging of amentoflavone in cross sections of *P. nudum* above-ground rhizomes [31].

Later, when liquid chromatography and suitable detectors became available, these methods became methods of choice for the separation and detection of 3′-8″-biflavones, especially when standards and suitable databases became commercially available. Usually, liquid chromatography is coupled with DAD or MS detectors [27]. The first report on separation and quantification of biflavones by liquid chromatography and spectrophotometric detector was reported in the early 1980s. In this publication, the authors separated four biflavones from ginkgo leaves, bilobetin, ginkgetin, isoginkgetin and sciadopitysin, using the LiChrosorb^®^ HPLC column and quantified them using a spectrophotometric detector. Today, the most commonly used detectors are mass detectors, but 3′-8″-biflavones have a strong signal at 330 nm, making the DAD detector in combination with standards a compelling, rapid, and accessible method for identification and quantification [178]. An example of a 330 nm chromatogram separating five 3′-8″-biflavones is presented in Figure 6a.

Over the past 20 years, MS-dependent imaging techniques have been developed to study the small-scale localization of compounds from complex biological systems. The most significant advances have been made with Assisted Laser Desorption/Ionization–Mass Spectrometry (MALDI-MS) imaging, which can be applied at both the tissue and single-cell level and provides information on the spatial distribution of specific molecules [209]. After tissue preparation and matrix application, the instrument acquires a series of mass spectra, each of which represents the profile of a specific region in the sample on a predefined x,y coordinate grid. This allows the gradients of individual analytes in the tissue to be visualized using specialized computer programs. The most commonly used ionization techniques besides MALDI are desorption electrospray ionization (DESI) and secondary ionization MS (SIMS). In the literature, ginkgo leaves in which 3′-8″-biflavones had been detected and localized were frequently used for method optimization [179,180]. Most of the available literature data showed that 3′-8″-biflavones, such as amentoflavone, bilobetin/sequioflavone, isoginkgetin/ginkgetin, sciadopytisyn, and methoxybilobetin, accumulate in the epidermis of ginkgo leaves [179,180]. The application of MALDI imaging in the study of the above-ground rhizome of *P. nudum* provided evidence for preferential accumulation of amentoflavone in cells of the chlorenchyma [31] (Figure 6b). The application of MALDI matrices to tissues sometimes complicates tissue preparation for imaging and can interfere with the native distribution of the metabolites under study. Therefore, a matrix-free laser desorption/ionization mass spectrometry method (LDI-MSI) was proposed in the study by Holscher et al. [210], which successfully detected amentoflavone in *Hypericum perforatum* pollen. MS-based imaging techniques provide valuable information not only on the presence of metabolites, but also on their localization, but are not widely used because of their high cost.

## 7. Conclusions and Further Directions

In recent years, 3′-8″-biflavones have become of interest as potential new compounds with pharmaceutical applications. Most studies have focused on their biological activity, with less information being available on their new natural sources and their role in plants. Most of the naturally occurring 3′-8″-biflavones have been elucidated as part of a larger screening study of natural products in specific plants, with few studies focusing specifically on biflavones. According to the available data, they are common in Pteridophyta, Gymnosperms, and Angiosperms, and so far, *G. biloba* and *Selaginella* sp. contain a variety of different 3′-8″-biflavones. Further studies to screen different species for the presence of 3′-8″-biflavones are needed and are likely to reveal their presence in more plant species and help to elucidate their role in plants and possible plant evolution. The content of 3′-8″-biflavones is highly dependent on the tissue type studied and other environmental factors, as shown by several studies on ginkgo. Although their exact role in plants is not clear, their localization in plants and tissues suggests their possible role in plant–environment interactions, especially biotic interactions, as they exhibit antimicrobial activity. Several studies suggest a possible role in the inhibition of photosynthesis, but more studies are needed to explain this statement and also other possible role in plants.

To study the role of 3′-8″-biflavones in plants, as well as their potential pharmaceutical use, efficient methods for their extraction and identification are being developed. Traditionally, most extraction methods for the extraction of 3′-8″-biflavones have been based on conventional methods such as organic solvent extraction, reflux extraction, percolation extraction, and Soxhlet extraction, but novel methods such as UAE, EAE, MAE, PLE, and new green solvents IL and DES are also being increasingly explored. In particular, extraction using environmentally friendly methods should be the focus in the future. It is challenging to conclude that a single method is suitable for extraction all 3′-8″-biflavones. When optimizing extraction methods, it is important to consider both the plant part used and the specific biflavone of interest, as their structures can vary significantly.

For the identification of new 3′-8″-biflavone structures, NMR was used, but for routine separation, identification, and quantification, especially when standards are available, HPLC coupled with MS or DAD is the method of choice. Great progress has also been made in the development of MALDI imaging methods for the identification and localization of 3′-8″-biflavones in tissues, particularly in *G. biloba* leaves. However, most of the studies performed are targeted analyses that are likely to miss some 3′-8″-biflavones. Therefore, more untargeted analyses using high-resolution mass spectrometry should be performed in the future to identify additional 3′-8″-biflavones.

## Figures and Tables

**Table 1:** Chemical formula of naturally occurring 3′-8″-biflavones.

	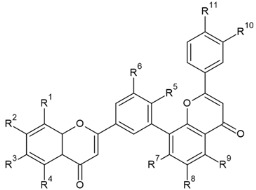											
Nr.	Compound	R^1^	R^2^	R^3^	R^4^	R^5^	R^6^	R^7^	R^8^	R^9^	R^10^	R^11^
**1.**	amentoflavone	H	OH	H	OH	OH	H	OH	H	OH	H	OH
**2.**	5′-hydroxyamentoflavone	H	OH	H	OH	OH	OH	OH	H	OH	H	OH
**3.**	sumaflavone	H	OH	H	OH	OH	H	OH	OH	OH	H	OH
**4.**	putraflavone (podocarpusflavone B)	H	OCH_3_	H	OH	OH	H	OH	H	OH	H	OCH_3_
**5.**	sequoiaflavone (7-*O*-methylamentoflavone)	H	OCH_3_	H	OH	OH	H	OH	H	OH	H	OH
**6.**	bilobetin	H	OH	H	OH	OCH_3_	H	OH	H	OH	H	OH
**7.**	ginkgetin	H	OCH_3_	H	OH	OCH_3_	H	OH	H	OH	H	OH
**8.**	isoginkgetin	H	OH	H	OH	OCH_3_	H	OH	H	OH	H	OCH_3_
**9.**	sciadopitysin	H	OCH_3_	H	OH	OCH_3_	H	OH	H	OH	H	OCH_3_
**10.**	4′,7″-di-*O*-methylamentoflavone	H	OH	H	OH	OCH_3_	H	OCH_3_	H	OH	H	OH
**11.**	7,4′,7″,4‴-*O*-methylamentoflavone	H	OCH_3_	H	OH	OCH_3_	H	OCH_3_	H	OH	H	OCH_3_
**12.**	podocarpusflavone A	H	OH	H	OH	OH	H	OH	H	OH	H	OCH_3_
**13.**	heveaflavone	H	OCH_3_	H	OH	OH	H	OCH_3_	H	OH	H	OCH_3_
**14.**	7,7″-di-*O*-methylamentoflavone	H	OCH_3_	H	OH	OH	H	OCH_3_	H	OH	H	OH
**15.**	7″-*O*-methylamentoflavone	H	OH	H	OH	OH	H	OCH_3_	H	OH	H	OH
**16.**	kayaflavone	H	OH	H	OH	OCH_3_	H	OCH_3_	H	OH	H	OCH_3_
**17.**	5′-methoxybilobetin	H	OH	H	OH	OCH_3_	OCH_3_	OH	H	OH	H	OH
**18.**	taiwanhomoflavone A	H	OCH_3_	CH_3_	OH	OCH_3_	H	OH	H	OH	H	OH
**19.**	oliveriflavone B	H	OH	CH_3_	OH	OCH_3_	H	OCH_3_	H	OH	H	OCH_3_
**20.**	oliveriflavone C	H	OH	CH_3_	OH	OCH_3_	H	OH	H	OH	H	OCH_3_
**21.**	amentoflavone 7,7″,4′,4‴-tetramethyl ether	OH	H	OCH_3_	H	OCH_3_	H	OCH_3_	H	OH	H	OCH_3_
**22.**	amentoflavone 7,7″-dimethyl ether	OH	H	OCH_3_	H	OH	H	OCH_3_	H	OH	H	OH
**23.**	7,4′,5″,7″,4‴-penta-*O*-methylamentoflavone	H	OCH_3_	H	OH	OCH_3_	H	OCH_3_	H	OCH_3_	H	OCH_3_
**24.**	amentoflavone 4′-methyl ether	OH	H	OH	H	OCH_3_	H	OH	H	OH	H	OH
**25.**	amentoflavone-7-methyl ether	OH	H	OCH_3_	H	OH	H	OH	H	OH	H	OH
**26.**	3‴-*O*-methylamentoflavone	H	OH	H	OH	OH	H	OH	H	OH	OCH_3_	OH
**27.**	sotetsuflavone	H	OH	H	OH	OH	H	OCH_3_	H	OH	H	OH
**28.**	amentoflavone-7,4′,7″,4‴-tetramethyl ether	H	OCH_3_	H	OH	OCH_3_	H	OCH_3_	H	OH	OCH_3_	H
**29.**	7,7″-dimethoxyamentoflavone	H	OCH_3_	H	OH	H	H	OCH_3_	H	OH	H	H
**30.**	7,7″,4′-tri-*O*-methylamentoflavone	H	OCH_3_	H	OH	OCH_3_	H	OCH_3_	H	OH	H	OH
**31.**	II-4″,I-7-dimethoxyamentoflavone	H	OCH_3_	H	OH	OH	H	OH	H	OH	OCH_3_	H
**32.**	amentoflavone-7″,4‴-dimethyl ether	H	OH	H	OH	OH	H	OCH_3_	H	OH	H	OCH_3_
**33.**	7,4′,7″,4‴-tetra-*O*-methylamentoflavone	H	OCH_3_	H	OH	OCH_3_	H	OCH_3_	H	OH	H	OCH_3_
**34.**	7,4′,7″-tri-*O*-methylamentoflavone	H	OCH_3_	H	OH	OCH_3_	H	OCH_3_	H	OH	H	OH
**35.**	7″,4‴-dimethylamentoflavone	H	OH	H	OH	OH	H	OCH_3_	H	OH	H	OCH_3_
**36.**	7,4′,4‴-trimethylamentoflavone	H	OCH_3_	H	OH	OCH_3_	H	OH	H	OH	H	OCH_3_

**Table 2:** Naturally occurring hydrogenation derivatives of 3′-8″ -biflavones.

	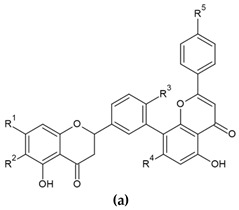	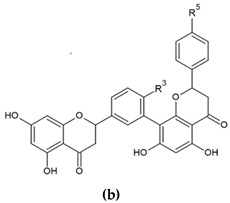				
**Nr.**	**Compound (a)**	**R^1^**	**R^2^**	**R^3^**	**R^4^**	**R^5^**
**37.**	(2S)-2,3-dihydroamentoflavone	OH	H	OH	OH	OH
**38.**	2,3-dihydrosciadopitysin	OCH_3_	H	OCH_3_	OH	OCH_3_
**39.**	2,3-dihydroisoginkgetin	OH	H	OCH_3_	OH	OCH_3_
**40.**	2,3-dihydro-7,7″-dimethoxyamentoflavone	OCH_3_	H	OH	OCH_3_	OH
**41.**	2,3-dihydro-6-methylginkgetin	OCH_3_	CH_3_	OCH_3_	OH	OH
**42.**	2,3-dihydroamentoflavone-7″,4‴-dimethyl ether	OCH_3_	H	OCH_3_	OH	OH
**43.**	(2*S*)-2,3-dihydro-4′-*O*-methylamentoflavone	OH	H	OCH_3_	OH	OH
**44.**	(2*S*,2″*S*)-2,3-dihydro-4′,4‴-di-*O*-methylamentoflavone	OH	H	OCH_3_	OH	OCH_3_
**45.**	2,3-dihydro-4‴-*O*-methylamentoflavone	OH	H	OH	OH	OCH_3_
		
	**Compound (b)**	**R^3^**			**R^5^**	
**46.**	(2*S*,2″*S*)-2,3,2″,3″-tetrahydroamentoflavone-4′-methyl ether	OCH_3_			OH	
**47.**	(2S,2″S)-2,3,2″,3″-tetrahydroamentoflavone	OH			OH	
**48.**	(2*S*,2″*S*)-2,3,2″,3″-tetrahydro-4′-*O*-methylamentoflavone	OCH_3_			OH	
**49.**	(2*S*,2″*S*)-2,3,2″,3″-tetrahydro-4′,4‴-di-*O*-methylamentoflavone (tetrahydroisoginkgetin)	OCH_3_			OCH_3_	

**Table 3:** Examples of the 3′-8″-biflavones named after the plants from which they were first isolated.

3′-8″-Biflavones	Plant Species
ginkgetin isoginkgetin bilobetin [33]	*Ginkgo biloba* L.
putraflavone [34]	*Putranjiva roxburghii*
sequoiaflavone [35]	*Sequoia sempervirens*
podocarpusflavone [36]	*Podocarpus* sp.
heveaflavone [37]	*Hevea braseliensis*
sciadopytisin [38]	*Sciadopitys verticillata*
oliveriflavone [39]	*Cephalotaxus oliveri*

**Table 4:** The list of the plants in which 3′-8″ biflavones have been identified.

Division	Genus	Species	Reported 3′-8″-Biflavone	Reference
Pteridophyta Ferns and fern allies	*Psilotum*	*nudum*	amentoflavone	[31]
	*Selaginella*	*bryopteris*	amentoflavone (2S)-2,3-dihydroamentoflavone (2″S)-2″,3″-dihydroamentoflavone (2S,2″S)-2,3,2″,3″-tetrahydroamentoflavone bilobetin sequoiaflavone heveaflavone sciadopitysin	[42]
		*delicatula*	amentoflavone	[43]
		*denticulata*	amentoflavone sotetsuflavone	[44]
		*doederleinii*	ginkgetin	[45]
			amentoflavone-4′-methyl ether amentoflavone-7-methyl ether	[46]
			podocarpusflavone A heveaflavone	[47]
			amentoflavone-7,7″,4′,4‴-tetramethyl ether	[45]
			7,4′,7″,4‴-tetra-O-methylamentoflavone amentoflavone 7,7″-di-O-methylamentoflavone heveaflavone	[48]
		*labordei*	amentoflavone	[49]
		*moellendorffii*	ginkgetin isoginkgetin	[50]
			kayaflavone podocarpusflavone A amentoflavone-7,4′,7″,4‴-tetramethyl ether	[51]
		*nothohybrida*	amentoflavone	[52]
		*rupteris*	amentoflavone	[53]
		*selaginoides*	amentoflavone	[44]
		*sinensis*	ginkgetin	[54]
			4′,7″-di-O-methylamentoflavone	[55]
		*stautoniana*	bilobetin	[56]
		*tamariscina*	sotetsuflavone heveaflavone	[57]
			sumaflavone amentoflavone taiwaniaflavone	[58]
			bilobetin	[59]
			2,3-dihydroamentoflavone 2″,3″-dihydroamentoflavone	[60]
		*uncinata*	(2S,2″S)-2,3,2″,3″-tetrahydroamentoflavone-4′-methyl ether (2″S)-2″,3″-dihydroamentoflavone-4′-methyl ether (2S)-2,3-dihydroamentoflavone-4′-methyl ether (2S,2″S)-tetrahydroamentoflavone (2S)-2,3-dihydroamentoflavone (2″S)-2″,3″-dihydroamentoflavone amentoflavone	[61]
		*willdenowii*	amentoflavone bilobetin 4′,7″-di-O-methylamentoflavone	[62]
	*Amentotaxus*	*yunnanensis*	sequoiaflavone sotetsuflavone sciadopitysin 2,3-dihydro-7,7″-dimethoxyamentoflavone 7,7″-dimethoxylamentoflavone	[63]
	*Araucaria*	*angustifolia*	ginkgetin	[64]
			bilobetin	[65]
	*Dacrydium*	*balansae*	amentoflavone sotetsuflavone 7″-O-methylamentoflavone	[66]
		*pierrei*	sotetsuflavone amentoflavone-4′,4‴,7,7″-tetramethyl ether	[67]
	*Decussocarpus*	*rospigliosii*	amentoflavone sequoiaflavone podocarpusflavone A podocarpusflavone B heveaflavone 7,7″-di-O-methylamentoflavone	[68]
	*Dioon*	*spinulosum*	sciadopitysin	[69]
	*Calocedrus*	*microleptic var. formosana*	amentoflavone	[70]
	*Cephalotaxus*	*drupacea*	ginkgetin	[71]
		*fortunei var. alpina*	ginkgetin	[72]
		*harringtonia*	ginkgetin	[73]
			bilobetin ginkgetin 7,7″,4′-tri-O-methylamentoflavone amentoflavone-7,7″,4′,4‴-tetramethyl ether 2,3-dihydro-6-methylginkgetin sciadopitysin	[74]
		*koreana*	ginkgetin amentoflavone bilobetin sciadopitysin 4′,7″-di-O-methylamentoflavone amentoflavone-7,7″,4′,4‴-tetramethyl ether 7,4′,7″,4‴-O-methylamentoflavone	[75]
		*oliveri*	oliveriflavone B oliveriflavone C	[76]
			sciadopitysin 7,4′,5″,7″,4‴-penta-O-methylamentoflavone	[39]
		*sinensis*	ginkgetin	[77]
		*wilsoniana*	taiwanhomoflavone A	[78]
	*Cunninghamia*	*lanceolata*	amentoflavone sequoiaflavone	[73]
	*Cupressocyparis*	*leylandii*	amentoflavone 7-O-methylamentoflavone podocarpusflavone A	[79]
	*Cupressus*	*funebris*	amentoflavone methylamentoflavone	[80]
		*sempervirens*		
		*glabra*		
		*goveniana*		
		*lusitanica*		
		*arizonica*		
		*torulosa*	amentoflavone podocarpusflavone A	[81]
	*Cycas*	*beddomei*	2,3-dihydro-4‴-O-methylamentoflavone 2,3,2″,3″-tetrahydroamentoflavone 2,3-dihydroamentoflavone	[82]
		*circinalis*	amentoflavone bilobetin isoginkgetin (2S,2″S)-2,3,2″,3″-tetrahydro-4′,4‴-di-O-methylamentoflavone (tetrahydroisoginkgetin) (2S,2″S)-2,3-dihydro-4′,4‴-di-O-methylamentoflavone (2S)-2,3-dihydro-4′-O-methylamentoflavone (2S,2″S)-2,3,2″,3″-tetrahydro-4′-O-methylamentoflavone	[83]
		*media*	ginkgetin	[84]
		*pectinata*	amentoflavone 2,3-dihydroamentoflavone	[85]
		*revoluta*	2,3-dihydroamentoflavone amentoflavone podocarpusflavone A (2S)-2,3-dihydroamentoflavone (2S,2″S)-2,3,2″,3″-tetrahydroamentoflavone	[77,83]
	*Chamaecyparis*	*obtusa*	sciadopitysin ginkgetin isoginkgetin podocarpusflavone A podocarpusflavone B 7,7″-O-dimethylamentoflavone bilobetin 7-O-methylamentoflavone sequoiaflavone podocarpusflavone A 7,7″-O-dimethylamentoflavone	[86]
	*Ginkgo*	*biloba*	amentoflavone bilobetin ginkgetin isoginkgetin sciadopytysin	[27]
			5′-methoxybilobetin	[32]
	*Juniperus*	*occidentalis*	amentoflavone	[87]
		*rigida*	amentoflavone	[88]
	*Microbiota*	*decussata*	amentoflavone 7-O-methylamentoflavone	[89]
	*Metasequoia*	*glyptostroboides*	sequoiaflavone podocarpusflavone A podocarpusflavone B isoginkgetin sciadopitysin amentoflavone 2,3-dihydroamentoflavone-7″,4‴-dimethyl ether amentoflavone-7″,4‴-dimethyl ether bilobetin ginkgetin 2,3-dihydroisoginkgetin 2,3-dihydrosciadopitysin	[90]
	*Nanuza*	*plicata*	amentoflavone 3′,8″-biisokaempferide	[91]
	*Ochna*	*schweinfurthiana*	amentoflavone	[92]
	*Ouratea*	*semiserrata*	amentoflavone podocarpusflavone A	[93]
	*Podocarpus*	*dacrydioides*	amentoflavone bilobetin sequoiaflavone podocarpusflavone A ginkgetin isoginkgetin podocarpusflavone B kayaflavone sciadopitysin	[94]
		*henkelii*	isoginkgetin 7,4′,7″,4‴-tetra-O-methylamentoflavone	[95]
		*elongatus*	isoginkgetin bilobetin	[96]
		*macrophyllus*	amentoflavone isoginkgetin	[97]
			podocarpusflavone A podocarpusflavone B	[77]
		*nagi*	amentoflavone-4′,4‴,7,7″-tetramethyl ether sciadopitysin	[98]
		*nakaii*	amenotoflavone podocarpusflavone A II-4″,I-7-dimethoxyamentoflavone heveaflavone	[99]
		*neriifolius*	amentoflavone podocarpusflavone A podocarpusflavone B isoginkgetin	[100]
		*imbricatus*	amentoflavone-7,7″-dimethyl ether heveaflavone	[101]
		*wallichiana*	isoginkgetin	[102]
	*Sciadopitys*	*verticillata*	amentoflavone podocarpusflavone A isoginkgetin	[81]
	*Sequoiadendron*	*giganteum*	amentoflavone podocarpusflavone A isoginkgetin	[81]
	*Taxodium*	*distichum var. distichum*	amentoflavone bilobetin podocarpusflavone A 7,4′,4‴-trimethylamentoflavone	Summarized by [11]
		*distichum var. mexicanum*	amentoflavone bilobetin	
	*Taxus*	*baccata*	ginkgetin sciadopitysin amentoflavone bilobetin	[81]
			podocarpusflavone A sequoiaflavone	[103]
		*chinensis*	ginkgetin	[104]
		*cuspidata*	ginkgetin	[105]
		*mairei*	ginkgetin ginkgetin	[106]
		*media*		
		*wallichiana*	ginkgetin	[107]
	*Thuja*	*plicata*	amentoflavone	[77]
		*orientalis*	amentoflavone	[108]
	*Torreya*	*nucifera*	amentoflavone bilobetin ginkgetin sciadopytisin	[109]
			4′,7″-di-O-methylamentoflavone kayaflavone	[74]
		*yunnanensis*	amentoflavone sotetsuflavone sciadopityisin	[63]
	*Retrophyllum*	*rospigliosii*	7,4′,7″,4‴-tetra-O-methylamentoflavone 7,4′,7″-tri-O-methylamentoflavone sciadopitysin 7,7″-di-O-methylamentoflavone podocarpusflavone A amentoflavone	[110]
	*Wollwmia*	*nobilis*	7,4′,7″,4‴-tetra-O-methylamentoflavone	[111]
Angiosperms Flowering plants	*Alchornea*	*glandulosa*	amentoflavone	[112]
		*triplinervia*	amentoflavone	[113]
	*Allanblackia*	*monticola*	amentoflavone podocarpusflavone A	[114]
	*Aletris*	*spicata*	amentoflavone	[115]
	*Androsace*	*umbellata*	amentoflavone sequioaflavone	[116]
	*Antidesma*	*bunius*	amentoflavone podocarpusflavone A	[117]
	*Antidesma*	*laciniatum*	amentoflavone	[118]
	*Amanoa*	*almerindae*	amentoflavone sequoiaflavone podocarpusflavone B	[119]
	*Biophytum*	*sensitivum*	amentoflavone	[120]
	*Byrsonima*	*crassa*	amentoflavone	[121]
		*intermedia*	amentoflavone	[122]
	*Caesalpinia*	*pyramidalis*	amentoflavone 5′- hydroxyamentoflavone podocarpusflavone A	[123]
	*Calophyllum*	*ferrugineum*	amentoflavone	[124]
		*flavoramulum*	amentoflavone	[125]
		*incrassatum*	amentoflavone	[126]
		*inophylloide*	amentoflavone	[127]
		*pinetorum*	amentoflavone	[128]
		*rivulare*	amentoflavone	[129]
		*symingtonianum*	amentoflavone	[126]
		*venulosum*	amentoflavone 2,3-dihydroamentoflavone	[130]
	*Campylospermum*	*elongatum*	7,7″-O-dimethylamentoflavone	[131]
		*calanthum*	amentoflavone sequoiaflavone podocarpusflavone B	[132]
		*mannii*	amentoflavone	[133]
	*Canarium*	*album*	amentoflavone	[134]
		*schwenfurthii*	amentoflavone	[135]
	*Capparis*	*spinosa*	gingetin	[136]
	*Casearia*	*clarkei*	amentoflavone	[137]
	*Celaenodendron*	*mexicanum*	amentoflavone bilobetin ginkgetin	[138]
			podocarpusflavone A podocarpusflavone B	[139]
	*Chrozophora*	*tinctoria*	amentoflavone	[140]
	*Cnestis*	*ferruginea*	amentoflavone	[141]
	*Cyperus*	*rotundus*	ginkgetin	[142]
			isoginkgetin	
	*Dorstenia*	*barteri*	amentoflavone	[143]
	*Drypetes*	*gerrardii*	amentoflavone	[144]
	*Elateriospermum*	*tapos*	amentoflavone ginkgetin podocarpusflavone B sequoiaflavone	[145]
	*Garcinia*	*bakeriana*	amentoflavone podocarpusflavone A 4‴-O-methylamentoflavone	[146]
		*brasiliensis*	amentoflavone podocrpusflavone A	[147]
		*brevipedicellata*	amentoflavone podocarpusflavone A	[148]
		*intermedia*	amentoflavone podocarpusflavone A	[149]
		*livingstonei*	amentoflavone	[150]
			podocarpusflavone A	[151]
		*madruno*	amentoflavone	[152]
		*merguensis*	amentoflavone	[153]
		*multiflora*	amentoflavone	[154]
		*subelliptica*	amentoflavone podocarpusflavone A garciniaflavone A garciniaflavone B garciniaflavone C garciniaflavone D	[155]
		*xanthochymus*	amentoflavone	[156]
	*Gaultheria*	*yunnanensis*	ginkgetin	[157]
	*Hevea*	*brasiliensis*	7″,4″-dimethylamentoflavone heveaflavone	[74]
	*Hypericum*	*connatum*	amentoflavone	[158]
		*perforatum*	amentoflavone	[159]
	*Hyeronima*	*alchorneoides*	amentoflavone	[160]
	*Lanaria*	*lanata*	amentoflavone	[161]
	*Lonicera*	*macranthoides*	amentoflavone 3‴-*O*-methylamentoflavone	[162]
	*Luxemburgia*	*nobilis*	amentoflavone	[163]
	*Lysimachia*	*christinae*	amentoflavone	[164]
	*Mangifera*	*indica*	amentoflavone	[165]
	*Ouratea*	*parviflora*	amentoflavone	[166]
		*ferruginea*	amentoflavone sequoiaflavone	[167]
		*multiflora*	amentoflavone podocarpusflavone A amentoflavone-7″,4‴-dimethyl ether heveaflavone	[168]
		*semiserrata*	amentoflavone podocarpusflavone A	[92]
		*sulcata*	amentoflavone	[169]
	*Ranunculus*	*ternatus*	kayaflavone	[170]
	*Rhus*	*pyroides*	amentoflavone	[171]
	*Rhus*	*succedanea*	amentoflavone	[154]
	*Speranskia*	*tuberculata*	amentoflavone	[172]
	*Struthiola*	*argentea*	amentoflavone	[173]
	*Viburnum*	*jucundum*	amentoflavone 2,3-dihydroamentoflavone	[174]
		*chinshanense*	amentoflavone	[175]
	*Zabelia*	*tyaihyonii*	amentoflavone	[176]

## Data Availability

Not applicable.
